# Inhibition Effect of Graphene Nanoplatelets on Electrical Degradation in Silicone Rubber

**DOI:** 10.3390/polym11060968

**Published:** 2019-06-03

**Authors:** Tao Han, Boxue Du, Jingang Su, Yu Gao, Yunqi Xing, Shengchen Fang, Chuanyang Li, Zhipeng Lei

**Affiliations:** 1 Key Laboratory of Smart Grid of Education Ministry, School of Electrical and Information Engineering, Tianjin University, Tianjin 300072, China; sujg1357@126.com (J.S.); fangscm@126.com (S.F.); 2 State Key Laboratory of Reliability and Intelligence of Electrical Equipment, Hebei University of Technology, Tianjin 300130, China; 3 State Grid Tianjin Electric Power Research Institute, Tianjin 300384, China; 4 Department of Electrical Engineering, University of Bologna, 40134 Bologna, Italy; lichuanyangsuper@163.com (C.L.); leizhipeng@163.com (Z.L.)

**Keywords:** electrical degradation, electrical tree, graphene, SIR, nanocomposites, inhibition

## Abstract

Silicone rubber (SIR) is widely used as an insulation material in high voltage cable accessories. Electrical tree is a typical electrical degradation and is easily initiated because of the distorted electric field. In this study, graphene nanoplatelets at contents of 0.001–0.010 wt % (0.00044–0.00436 vol %) were added into SIR to improve the electrical tree inhibiting ability. Scanning electron microscopy, conductivity and surface potential decay tests were conducted to analyze the characteristics of graphene/SIR nanocomposites. The typical electrical treeing experiment was employed to observe the electrical tree inhibition of graphene in SIR. The results show that graphene nanoplatelets were well dispersed in SIR. The conductivity was higher after the addition of graphene nanoplatelets, and the trap distribution was affected by graphene nanoplatelets. The tree was changed from a bush-branch structure to a bush structure after the addition of graphene. Tree inception voltage improved and reached the highest mean value at 0.003 wt %. The tree length was inhibited at 0.001 to 0.007 wt % and the lowest tree length occurred at 0.005 wt %.

## 1. Introduction

Silicone rubber (SIR) is usually employed as insulation in both alternating current (AC) and direct current (DC) power cable accessories, forming a multilayer insulation with cross-linked polyethylene (XLPE), polypropylene (PP) or other polymers [1,2,3]. Given the distortion of electric field and manufacturing defects, electrical degradation is easily initiated in SIR [4,5,6]. As a typical electrical degradation, the electrical tree initiation and growth have been widely researched since it was first identified [7,8,9,10].

Nanodielectrics with filler contents of several wt % have been proven to have a high electrical tree-inhibiting ability [11,12,13,14]. Traditional inorganic nanoparticles have also been used to improve the tree resistance of SIR, including silica (SiO_2_) and alumina (Al_2_O_3_) [15,16,17]. With a high enough content (lower than 10 wt %) of nanoparticles, trees are considerably inhibited [15,16,17]. The multi-core model proposed by Tanaka et al. explained the inhibition effect of nanoparticles on electrical trees [18]. According to this model, the multilayer structure around the nanoparticle produces a number of traps, which capture the injected space charges and uniform the electric field in nanocomposites. It has been verified that the particles with higher dielectric strength will inhibit tree growth and act as tree barriers, as reviewed by Danikas et al. [19,20]. From this perspective, one effective method to inhibit electrical trees is to create a huge interface between the nanofillers and matrix. It can be inferred that the optimal content of nanofillers with a two-dimensional (2D) structure may be much lower than the traditional nanofillers because of the high specific surface area (SSA).

Graphene is a typical 2D structure with a giant specific surface area. The effects of graphene nanoplatelets on SIR properties have been researched [21,22,23,24]. In these studies, several wt % of graphene or graphene oxide nanoplatelets were added into SIR to improve the thermal or mechanical properties. However, the conductivity of SIR was increased by several orders of magnitude with the content of this fillgrade of graphene, which is bad for an insulation material [21,22]. For the insulation properties, it was found that a content lower than 0.01 wt % increases trap levels in graphene/low-density polyethylene (LDPE) nanocomposites [25]. With a higher content of 0.01%, the positive effect disappears [25]. Graphene at a content of 0.005 wt % could markedly suppress the space charge injection and accumulation in LDPE [26]. In the treeing process, the trap level and space charge also play important roles [27]. However, the effect of graphene on electrical trees in SIR has not yet been reported.

In this work, graphene/SIR nanocomposites were prepared with filler contents of 0, 0.001, 0.003, 0.005, 0.007 and 0.010 wt %. Scanning electron microscopy (SEM), conductivity and trap distribution were tested. The electrical treeing experiment was conducted and the inhibition effect of graphene was discussed.

## 2. Materials and Methods

### 2.1. Preparation of Graphene/SIR Nanocomposites

High temperature vulcanized methyl vinyl SIR (MVQ 110-2) was provided by Dongjue Silicone Group Co. Ltd. (Nanjing, China). Its average molecular weight was 65 × 10^4^ g/mol, and the content of vinyl groups was 0.13–0.20 mol %. The graphene used for the sample was purchased from Tanfeng Graphene Technology Co., Ltd. (Nanjing, China). The diameter of graphene nanoplatelets was 0.5–5 μm, the thickness was 0.5–3.0 nm, with 1–2 layers and an SSA of 1000–1217 m^2^/g. Before mixing, a surface treatment was applied on the graphene.

Zhang et al. reported that graphene modified with vinyltrimethoxysilane exhibited improved dispersing and banding properties with SIR [22]. Thus, vinyltrimethoxysilane (VTMS, Henan Tianfu Chemical Co., Ltd., Zhengzhou, China) was used as the surfactant for surface treatment. The procedures were as follows: In the first step, the graphene was dispersed into ethanol for 30 min. Then, magnetic stirring was conducted for 10 h at 60 °C. The mass ratio of the VTMS to graphene was 1.5:1 [22]. Then, magnetic stirring was carried out for 10 h at 60 °C. Subsequently, the mixture was filtered and washed several times with methanol. After this, the surface-modified graphene was placed into a vacuum oven at 80 °C for 12 h to be dried.

The weight content of graphene in nanocomposites was 0, 0.001, 0.003, 0.005, 0.007 and 0.010 wt % (0, 0.00044, 0.00132, 0.00219, 0.00307 and 0.00436 vol %, respectively). The required graphene was weighed by a microbalance with a sensitivity of 0.01 mg (CPA225D, Sartorius AG, Göttingen, Germany). A twin-screw continuous mixer was employed to mix the graphene and SIR, with a rotation speed of 40 rpm at room temperature for 15 min. Then 2,5-dimethyl-2,5-di(tert-butylperoxy) hexane (DBPMH, Dongguan Tengkai Rubber and Plastic Technology Co., Ltd., Dongguan, China) was added into the mixture as the vulcanizing agent at 1.5 phr. After the mixing process, the compounds were placed into the moulding in the plate vulcanizing machine (XLBD-100T, Nanjing Mixer Industrial Co. Ltd. Nanjing, China) at a temperature of 165 °C and pressure of 10 MPa for 10 min [21]. After this, the vulcanized specimen with the dimension of 90 × 90 × 3 mm was placed in an oven at 180 °C for 3 h for the second step of vulcanization.

### 2.2. Morphology Analysis of Nanocomposites

A high-resolution scanning electron microscope (SEM, FEI SCIOS, Thermo Fisher Scientific, Waltham, Massachusetts, NY, USA) was employed to observe the cross-section of the graphene/SIR nanocomposites. The samples were cryo-fractured in liquid nitrogen and the surface of the cross-section was painted with platinum before scanning.

### 2.3. Conductivity Measurement

DC conductivity was tested on a typical three-electrode system as in [28]. The sample for the DC conductivity test had a radius of 1.25 cm and thickness of 700 μm. Given the increase of conductivity with temperature and electric field [29], the conductivity under fixed temperature and electric field was employed here to analyze the effect of graphene. In this test, the voltage was 3000 V and temperature was 20 °C. The ampere meter for the current test was a Keithley 6517b (Keithley Instruments, Cleveland, OH, USA).

After 10,000 s, the conductivity was calculated as follows:

(1) $$\sigma =\frac{I}{U}\cdot \frac{l}{\pi r^{2}},$$

where *I* is the current in A, *U* is the applied voltage in V, *l* is the thickness of sample in m and *r* is the radius of sample in m.

### 2.4. Surface Potential Decay and Trap Distribution Measurement

To calculate the trap distribution in SIR, surface potential decay (SPD) measurement was used. The measurement setup was shown in our previous work [25]. The sample for the SPD test was round in shape with a radius of 20 mm and thickness of 300 μm. Before the test, the surface of the sample was charged by DC corona. The voltage applied on the needle electrode was −7 kV DC. After the 15-min charging process, the sample was moved to the probe of the electrostatic voltmeter (Trek 347-3HCE, TREK, Inc., New York, NY, USA). The probe was positioned 3 mm above the center of the sample surface. The decay of surface potential was recorded by an electrostatic voltmeter. The temperature of the sample was maintained at 20 °C, and the relative humidity was controlled at 25%.

The potential decay rate is related to the trap distribution in samples. The trap energy and density can be calculated by these three equations [25,30]:

(2) $$E_{T}=k_{B}T\ln(v_{\mathrm{ATE}}t),$$

(3) $$v_{\mathrm{ATE}}=\frac{k_{B}T}{h},$$

(4) $$N_{T}=\frac{4\varepsilon_{0}\varepsilon_{r}}{qk_{B}TL^{2}}\left|t\frac{dU_{s}}{dt}\right|,$$

where *E*_T_ is the trap energy in eV; *T* is the temperature in K; *v*_ATE_ is the attempt to escape frequency in s^–1^; *k*_B_ is the Boltzmann’s constant in eV/K; *h* is the Planck constant in eV·s; *N*_T_ is the trap density in eV^–1^m^–3^; *ε*_0_ and *ε_r_* are the permittivity of vacuum and the relative permittivity of SIR; *q* is the elementary charge in C; *L* is the sample thickness in m; *U_s_* is the surface potential in V and *t* is the decay time in s.

### 2.5. Electrical Treeing Experiments

**Figure 1:**
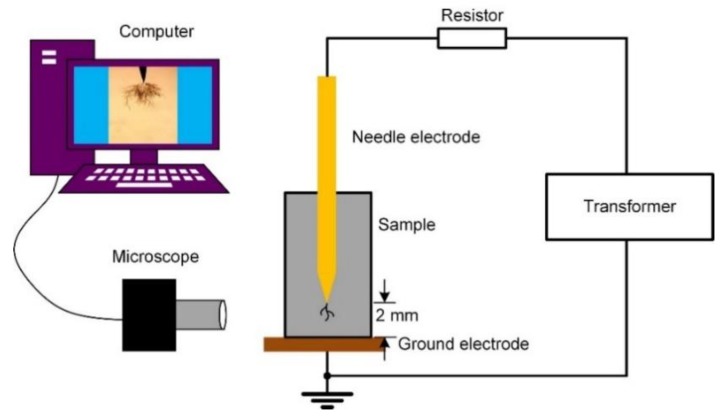
Treeing experiment setup.

The treeing test setup is shown in Figure 1. The size of sample was 40 × 20 mm, with a thickness of 3 mm. During the test, high voltage was applied by a transformer (YDJ/TDM-2/50, Sanxin Electrical Equipments, Wuhan, China). The frequency of the AC voltage was 50 Hz and the root-mean-square (RMS) varied from 5 to 10 kV. The voltage increase rate was 1 kV/s. A needle electrode was inserted into samples as the high voltage electrode. The curvature radius of the needle tip was 3 μm. The needle was chosen using a microscope before it was employed. The distance from the needle tip to the edge of the sample was set to 2 mm. A copper foil was attached to the bottom of sample as the ground electrode during the test. A resistor was connected in the test circuit for protection of the transformer. The treeing process was recorded by a digital microscope (XDC10A, KENI, Shenzhen Sangnond Technology Co., Ltd., Shenzhen, China). The treeing time was set to 1 h with each voltage. The temperature was 20 °C.

## 3. Results

### 3.1. Morphology and SEM

**Figure 2:**
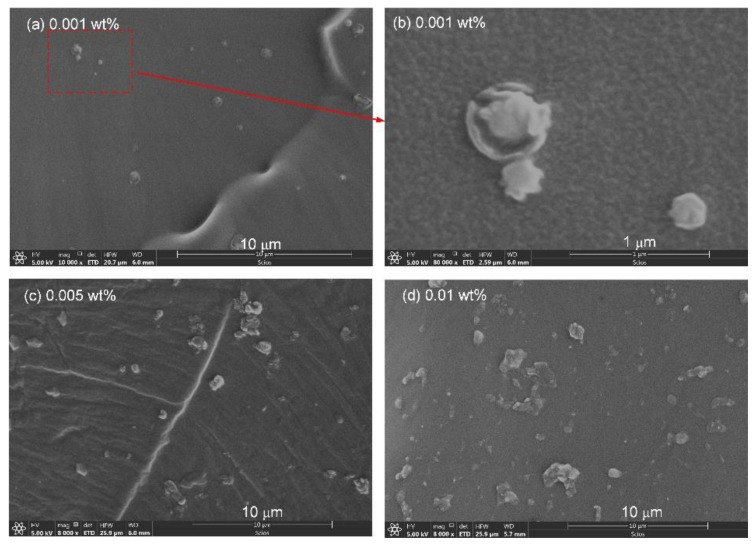
Scanning electron microscopy (SEM) images of different samples: (**a**) SEM of nanocomposites with graphene content of 0.001 wt %; (**b**) Enlarged SEM of (**a**); (**c**) SEM of nanocomposites with a graphene content of 0.005 wt % and (**d**) SEM of nanocomposites with a graphene content of 0.010 wt %. Graphene nanoplatelets with a lighter color can be found in each figure.

The graphene nanoplatelets distribution in the samples for all graphene contents were analyzed with SEM. The SEM pictures of samples with 0.001, 0.005 and 0.010 wt % are shown in Figure 2. Figure 2a,b shows the SEM images of nanocomposites with 0.001 wt %. The graphene nanoplatelets dispersed well in this sample. It should be noted that the orientation of graphene nanoplatelets would be random because no orientation inducement was employed in the preparation. Then, graphene nanoplatelets vertical to the cross-section would not be observed in SEM because of the thickness of 0.5–3 nm. An enlarged region from Figure 2a is shown in Figure 2b. Figure 2c,d shows the dispersion in nanocomposites with 0.005 and 0.010 wt %. With the increase in graphene content, the distance between the graphene nanoplatelets decreased. In Figure 2d, some of the graphene nanoplatelets were bridged at the content 0.010 wt %.

### 3.2. DC Conductivity of Graphene/SIR Nanocomposites

**Figure 3:**
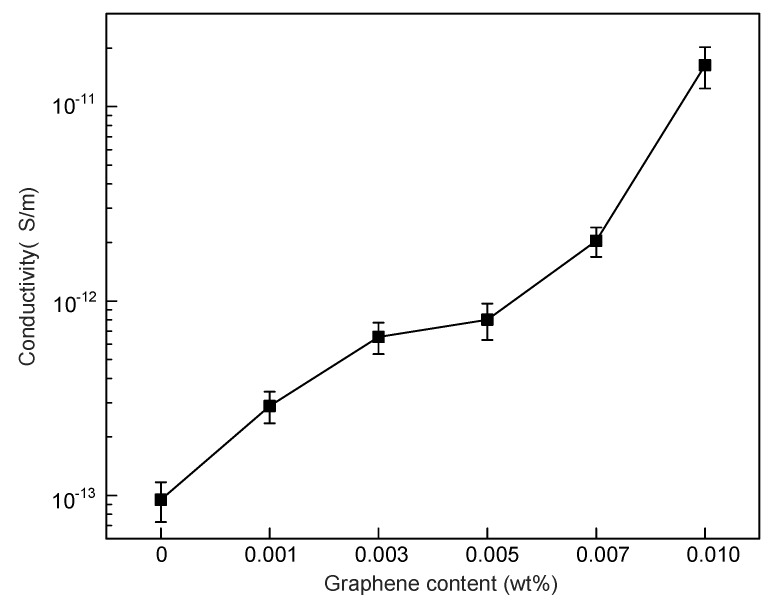
Conductivity of graphene/silicone rubber nanocomposites.

The conductivity of graphene/SIR nanocomposites is shown in Figure 3. The data are calculated from five test results at each content and the standard deviations are shown in Figure 3. With the content increasing from 0 to 0.005 wt %, the conductivity of the nanocomposites was increased by 7.06 × 10^−13^ S/m. From 0.005 to 0.010 wt %, a sharp increase (1.55 × 10^−11^ S/m) in conductivity occurred, potentially caused by the shorter distance between graphene nanoplatelets at higher graphene contents.

### 3.3. SPD Characteristics and Trap Distribution of Graphene/SIR Nanocomposites

**Figure 4:**
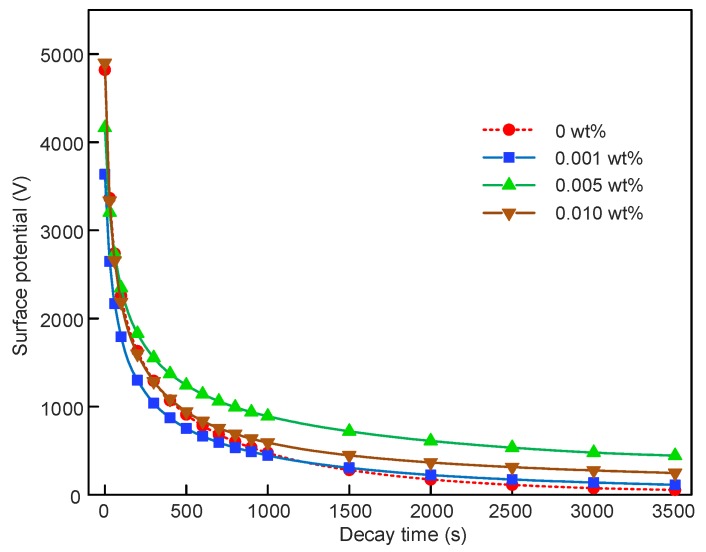
Surface potential decay in graphene/silicone rubber nanocomposites.

The potential decay in graphene/SIR nanocomposites is related to the charge migration from SIR bulk [31,32]. During the SPD tests, five samples were employed for each content. The typical results at 0, 0.001, 0.005 and 0.010 wt % from the SPD test are shown in Figure 4. With the graphene content increasing from 0 to 0.010 wt %, the decay rate decreased firstly and then increased.

**Figure 5:**
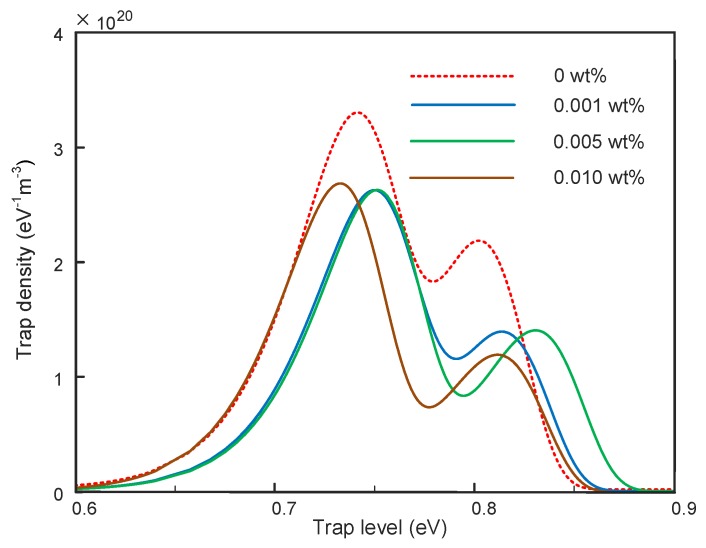
Trap distribution in graphene/silicone rubber nanocomposites.

The trap distribution in the nanocomposites can be calculated according to Equations (2)–(4). The relative permittivity used here is shown in Table 1. As shown in Figure 5, the results calculated from data in Figure 4 are employed here to simplify the illustration. It can be found that the trap distribution characteristics were changed by the addition of graphene. There were two peaks in the trap distribution curve, defined as shallow and deep trap levels. As shown in Figure 5, both the shallow and deep trap levels shifted to the right after the addition of graphene at 0.001 and 0.005 wt %. This indicated that it was harder for charges to detrap in nanocomposites with 0.001 and 0.005 wt %, whereas in nanocomposites with 0.010 wt % graphene, the shallow trap level was lower than that of 0 wt % and the deep trap level was approximate to that of 0 wt %. The trap density was also affected by the addition of graphene. We found that the density of the shallow trap decreased from 3.4 × 10^20^ eV^–1^m^–3^ to lower than 2.7 × 10^20^ eV^–1^m^–3^ and the density of deep trap decreased from 2.2 × 10^20^ eV^–1^m^–3^ to lower than 1.7 × 10^20^ eV^–1^m^–3^ after the addition of graphene.

### 3.4. Electrical Tree in Graphene/SIR Nanocomposites

#### 3.4.1. Tree Structure in Graphene/SIR Nanocomposites

**Figure 6:**
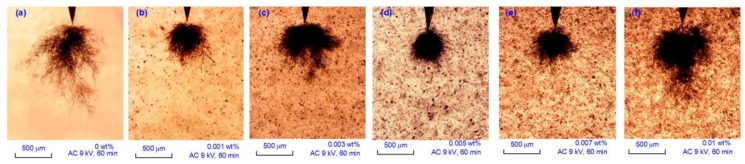
Tree structures at different graphene contents. (**a**) Bush-branch tree at 0 wt %; (**b**–**f**) bush trees at 0.001–0.010 wt %.

During the experiment, two different tree structures were observed, defined as bush-branch tree and bush tree. As shown in Figure 6a, the bush-branch tree had a combined structure—there was a bush structure near the needle tip and a branch area out of the bush structure. In the bush area, all the tree channels interlaced with each other, forming a totally black area. In Figure 6b–f, all the trees are the bush structure. In the undoped SIR sample, all trees were the bush-branch structure at 60 min. For graphene content from 0.001 to 0.007 wt %, all the trees were typical bush structure; at the content of 0.010 wt %, the bush tree was much longer than the others.

#### 3.4.2. Tree Inception Voltage in Graphene/SIR Nanocomposites

To observe the inhibition effect of graphene, the tree inception voltage was tested. The RMS of the AC voltage was set to 5–10 kV. In the test, the voltage was increased in 0.2-kV steps and was maintained for 30 s at each step to check if the tree was triggered or not. If an electrical tree with a length of 20 μm was observed, the RMS was recorded as the tree inception voltage. Ten samples were tested for each graphene content.

**Figure 7:**
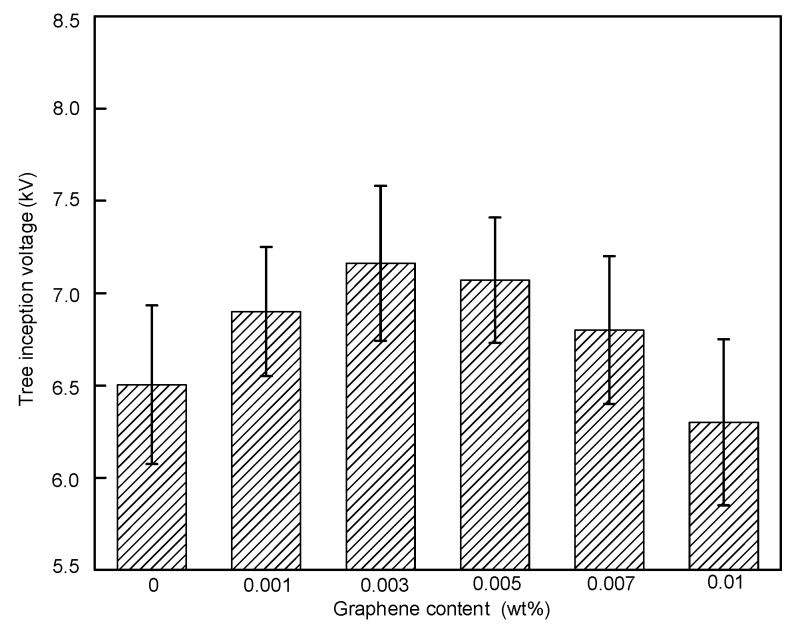
Tree inception voltage with different graphene contents.

The average inception voltages from 10 tests and the standard deviations are shown in Figure 7. The average inception voltage with 0 wt % was 6.5 kV. With the contents ranging from 0.001 to 0.007 wt %, the inception voltage increased to around 7.0 kV. When the content increased to 0.010 wt %, the average inception voltage decreased to 6.3 kV. The addition of graphene increased the inception voltage at contents lower than 0.007 wt % and decreased the inception voltage at 0.010 wt %.

#### 3.4.3. Tree Growth in Graphene/SIR Nanocomposites

**Figure 8:**
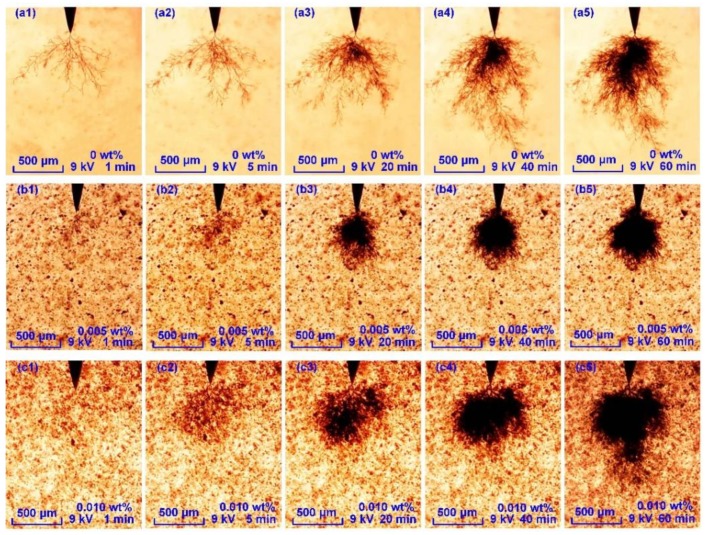
Tree growth process in graphene/silicone rubber (SIR) nanocomposites with different contents. (**a1**–**a5**) Tree growth in undoped SIR; (**b1**–**b5**) tree growth in nanocomposites with 0.005 wt % and (**c1**–**c5**) tree growth in nanocomposites with 0.010 wt %.

The treeing process was recorded using a microscope and computer. Typical tree growth processes in samples with different graphene contents are shown in Figure 8.

As shown in Figure 8a1–a5, the tree in the undoped SIR sample displayed the branch structure in the first 20 min. Then the bush area appeared near the needle tip. All the branch channels continued growing simultaneously. At the treeing time of 60 min, the typical bush-branch tree formed.

As shown in Figure 8b1–b5, there were many tiny branches after the tree inception. At the same treeing time in the first 5 min, the bush tree size was much smaller than the bush-branch tree. With the increase in treeing time, the bush area became darker and the size continued to grow. However, there was no obvious length growth for this bush tree from 20 to 60 min.

As shown in Figure 8c1–c5, a typical bush tree formed in the first 40 min in the sample with 0.010 wt %. After this, an obvious growth of tree length occurred, making the tree much longer than that of the 0.005 wt %.

**Figure 9:**
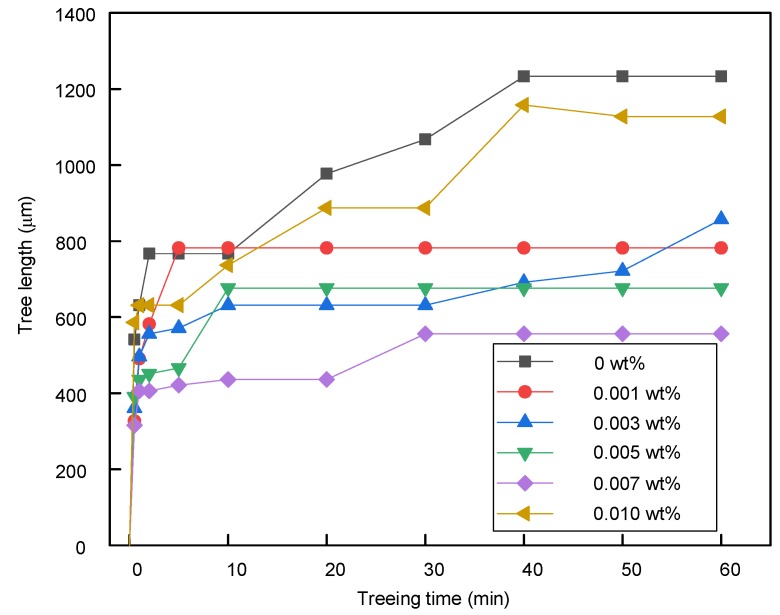
Tree growth process at different graphene contents.

The tree length of a typical tree without breakdown at different times was calculated as shown in Figure 9. Figure 9 shows that a fast length growth process occurred in the first three minutes. After this stage, the growth in undoped SIR and graphene/SIR nanocomposites showed different tendencies. In the undoped SIR, the tree length continued growing at a higher speed and reached 1200 μm at 60 min. In the nanocomposites with graphene contents ranging from 0.001 to 0.007 wt %, there was no obvious growth in tree length from 10 to 60 min. This shows that the tree length growth was inhibited by the graphene at these contents. In the sample with 0.010 wt %, the tree length growth process was similar to that of undoped SIR.

**Figure 10:**
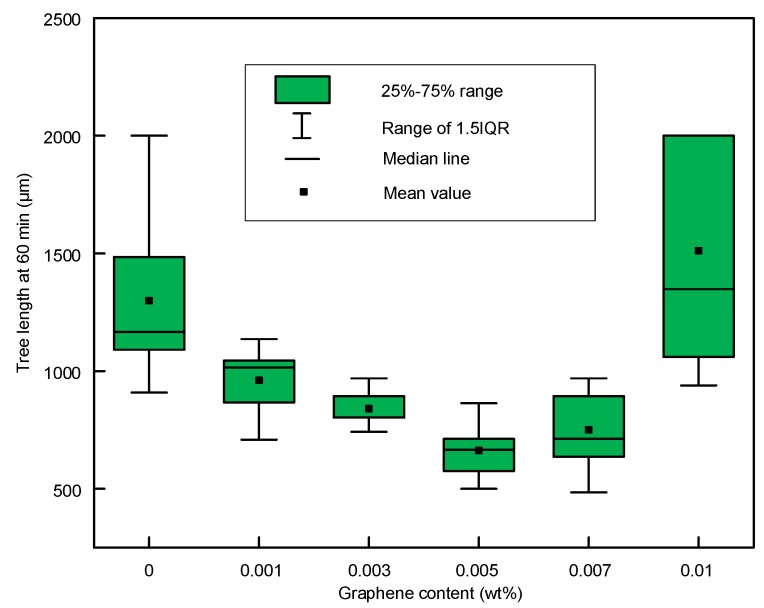
Tree length at 60 min with different graphene contents.

During the experiments, nine samples of each graphene content were tested for 60 min. The tree length at 60 min was calculated to analyze the inhibition effect of graphene. If the breakdown occurred within 60 min, the length was calculated as 2 mm. In the whole experiment, breakdown only occurred in samples with 0 and 0.010 wt %. The box-plot of tree length at 60 min is shown in Figure 10. We found a nonlinear change in the average tree length. From 0 to 0.005 wt %, the tree length decreased. From 0.005 to 0.010 wt %, the tree length increased. The tree lengths in the samples with 0 and 0.010 wt % were more scattered. This was because breakdown occurred in some of these samples. 1 and 3 breakdowns occur in nine trees at 0 and 0.010 wt %, respectively.

## 4. Discussion

### 4.1. Tree Inception

Under AC voltage, there is an electric field with altering direction. According to the theory proposed by Tanaka et.al., it is believed that there is an electron injection-extraction process near the needle tip [33]. Given the existence of traps in undoped SIR, some of these electrons are captured by traps near the needle tip after the injection. After the changing of electric field direction, some of these injected electrons will be extracted. In this repetitive injection and extraction process, collisions with molecular chains occur. If the electrons gain sufficient energy, this collision will break the molecular chains near the needle tip, producing lower molecular weight products and gas [33]. A partial discharge occurs if this region is big enough and the first tree channel will be triggered.

In the case of nanocomposites, the trapping-detrapping process is similar to what occurs in undoped SIR. When the electrons are injected into the sample, they will be captured by the traps around needle tip induced by the interface between SIR and graphene [26,34]. However, with graphene contents lower than 0.007 wt %, the trap levels of graphene/SIR nanocomposites are higher than undoped SIR. As a result, the trapped electrons need more energy to detrap in these nanocomposites. A higher voltage is needed for the material degradation and the occurrence of partial discharge. As a result, tree inception voltage increases obviously in graphene/SIR nanocomposites, compared with an undoped SIR sample. However, the shorter distance between graphene nanoplatelets in samples at 0.010 wt % lower the trap levels, as shown in Figure 6. This indicates that the trapped electrons need lower energy to escape and gain sufficient energy [26,34]. As a result, there is a lower inception voltage in nanocomposites at 0.010 wt %.

### 4.2. Tree Growth

**Figure 11:**
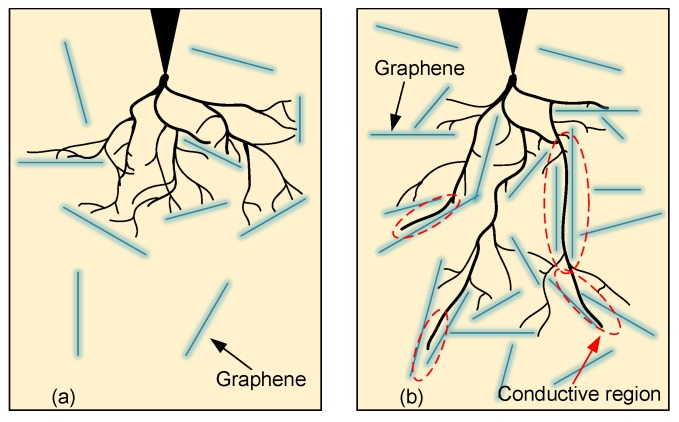
Effect of graphene nanoplatelets in the treeing process; (**a**) Inhibition effect of graphene and the formation of the bush tree; (**b**) Conductive regions at high graphene contents and their acceleration effect on tree growth.

According to the model proposed by Danikas et al. [20], the tree growth is inhibited by the particles in nanocomposites. In graphene/SIR nanocomposites, there are huge surface areas between the SIR and graphene nanoplatelets, even at low graphene content. As shown in Figure 11a, the graphene nanoplatelets are well dispersed in silicone rubber. In this condition, the graphene nanoplatelets will act as barriers during the treeing process. The tree channels will grow towards the graphene because of the electric field distortion and then grow along the interfaces. Because of the 2D structure and different orientations of graphene nanoplatelets, a bush tree forms rapidly after the inception and the tree length growth is inhibited, which is consistent with the treeing process in Figure 8b1–b5.

However, the shorter distance between the nanoplatelets lower the dielectric strength in some regions inside the nanocomposites with higher content [34]. Because of the high conductivity of graphene nanoplatelets, these regions between the adjacent nanoplatelets may be conductive. The tree channels will go through these regions fast, as shown by the red circles in Figure 11b. With the increase of graphene content, the occurrence probability of the conductive regions becomes higher. This will lead to low degradation resistance, longer tree lengths and even breakdown at 0.010 wt %.

## 5. Conclusions

Graphene nanoplatelets and SIR were employed to prepare nanocomposites with graphene content lower than 0.010 wt %. SEM, conductivity and trap distribution characteristics were tested to examine the nanocomposites.

The results showed that the conductivity of SIR can be increased by the addition of graphene nanoplatelets. The trap distribution in SIR changes with the addition of graphene nanoplatelets. The shallow and deep trap levels in samples increase in nanocomposites with content ranging from 0.001 to 0.007 wt %.

The electrical degradation was inhibited by the addition of graphene nanoplatelets at low contents. The average tree inception voltage increased after the addition of graphene nanoplatelets at contents of 0.001 to 0.007 wt %. The tree structure in undoped SIR was a bush-branch tree, which changed to the bush tree structure in graphene/SIR nanocomposites. The tree length in graphene/SIR nanocomposites was inhibited at contents of 0.001 to 0.007 wt % and increased at 0.010 wt %.

## Figures and Tables

**Table 1:** Relative permittivity results at different graphene contents.

Graphene Content (wt %)	0	0.001	0.003	0.005	0.007	0.010
Relative permittivity	3.16	3.05	3.18	3.19	3.22	3.28
